# *TERSE/PROLIX* (*TRPX*) – a new algorithm for fast and lossless compression and decompression of diffraction and cryo-EM data

**DOI:** 10.1107/S205327332300760X

**Published:** 2023-09-25

**Authors:** Senik Matinyan, Jan Pieter Abrahams

**Affiliations:** a Biozentrum, University of Basel, Basel, Switzerland; b Laboratory of Nanoscale Biology, Paul Scherrer Institute, Villigen, Switzerland; Czech Academy of Sciences, Czech Republic

**Keywords:** compression, *TERSE/PROLIX*, *TRPX*, lossless, diffraction data, cryo-EM data, lossless data compression

## Abstract

High-throughput data collection in crystallography poses significant challenges in handling massive amounts of data. Here, *TERSE/PROLIX* (or *TRPX* for short) is presented, a novel lossless compression algorithm specifically designed for diffraction data. The algorithm is compared with established lossless compression algorithms implemented in *gzip*, *bzip2*, CBF (crystallographic binary file), *Zstandard*(*zstd*), *LZ4* and HDF5 with *gzip*, *LZF* and *bitshuffle*+*LZ4* filters, in terms of compression efficiency and speed, using continuous-rotation electron diffraction data of an inorganic compound and raw cryo-EM data. The results show that *TRPX* significantly outperforms all these algorithms in terms of speed and compression rate. It was 60 times faster than *bzip2* (which achieved a similar compression rate), and more than 3 times faster than *LZ4*, which was the runner-up in terms of speed, but had a much worse compression rate. *TRPX* files are byte-order independent and upon compilation the algorithm occupies very little memory. It can therefore be readily implemented in hardware. By providing a tailored solution for diffraction and raw cryo-EM data, *TRPX* facilitates more efficient data analysis and interpretation while mitigating storage and transmission concerns. The C++20 compression/decompression code, custom TIFF library and an *ImageJ*/*Fiji* Java plugin for reading *TRPX* files are open-sourced on GitHub under the permissive MIT license.

## Abstract 2

This article presents a fast and lossless algorithm for compressing diffraction data, achieving up to 85% reduction in file size while processing up to 2000 512 × 512 frames s^−1^. This breakthrough in compression technology is a significant step towards more efficient analysis and storage of large diffraction data sets.

## 1. Introduction

Universal access to exponentially growing data has made efficient data storage and processing crucial for transformative science (Hill *et al.*, 2016 ▸; Tolle *et al.*, 2011 ▸). In crystallography, the emergence of hybrid pixel detector technology has led to a significant increase in the amount of data generated per data collection session, producing exponentially growing volumes of diffraction data (Paton *et al.*, 2021 ▸; Tate *et al.*, 2016 ▸). Because these detectors are so fast and have no readout noise, fine phi-slicing and high frame rates allow more accurate data, with many pixels having values close to zero. However, the resulting high acquisition rates of diffraction data are out­pacing the data transfer capabilities to local storage, presenting a significant challenge (Kieffer *et al.*, 2018 ▸; Stroppa *et al.*, 2023 ▸). Additionally, the size of current data sets poses challenges for transferring, sharing and collaborating effectively, leading to increased operational costs, reduced experimental throughput, and potentially lost scientific information due to inefficiencies in data handling.

To address these challenges, there is an urgent need for robust, more efficient diffraction data compression that is lossless and fast enough to keep up with the high frame rates of modern detectors. In this context, we present the *TERSE/PROLIX* algorithm (*TRPX* for short), a novel compression method specifically designed for diffraction data. Initial tests have shown that it can compress integral data to at least 15% of the initial size and handle up to 2000 electron diffraction frames per second (512 × 512 16-bit pixels), using a single core on a modern laptop (we used Apple’s M1 Max processor for testing purposes). By providing a tailored solution for diffraction data, *TRPX* mitigates storage and transmission concerns. This algorithm has the potential to significantly improve the handling and long-term preservation of high-throughput diffraction data, facilitating scientific discoveries and accelerating the pace of transformative science.

## 2. *TRPX* compression algorithm

Diffraction data frames typically consist of a large number of grayscale pixels with integral values and a high dynamic range. At lower resolutions, the pixels tend to have higher values in Bragg peaks, while between the Bragg peaks and at higher resolutions, they have lower values. Thus, diffraction data frames are spatially correlated. By leveraging these inherent properties, *TRPX* performs lossless, efficient and fast compression of integral diffraction data frames and other integral grayscale data. The algorithm was specifically designed for speed, but we found it to be also superior in reducing file sizes.

The algorithm was devised for lossless compression of diffraction and imaging data of any type (X-ray, electron, neutron). It accepts grayscale pixel values with a very high dynamic range (up to 64 bits). Data can be extracted as integer or floating-point types, but need to be encoded as integers before compression. It will compress any grayscale data, including cryo-EM data, quickly and without loss of precision. The compression rate is determined by the number of bits required to encode the majority of pixel values for images in which the pixels are locally correlated.

### 2.1. Compression scheme

The *TRPX* algorithm uses a run-length encoding approach, which compresses data by identifying repeated patterns (Robinson & Cherry, 1967 ▸). Unlike most other run-length encoding algorithms, it requires just a single pass through the data, analogous to an early algorithm for compressing diffraction data (Abrahams, 1993 ▸). Implemented in C++20, it can easily be linked into other programs, which may be written in computer languages other than C++. The code creates a ‘Terse’ object that compresses data with a pixel depth of up to 64 bits. The data can be stored in a standard C++ data container, or can be provided to the ‘Terse’ object as a memory location or a stream of raw data, together with the number of pixels. A ‘Terse’ object may hold a stack of same-sized images. The algorithm compresses data quickly and efficiently by identifying patterns in a single pass using primitive processor operators. The resulting ‘Terse’ object can be written as a byte-stream, which is independent of the endianness of the machine, ensuring that both big- and little-endian machines produce identical files. Because it can also be appended to existing files, it can be embedded in other data formats that may have specific header information, by replacing the raw data section. A .trpx file has a small XML header that contains essential metadata required for unpacking, and that can easily be extended for specific use cases. By default, images are assumed to be two-dimensional and square, but this can be overridden by specifying image dimension parameters, which are then included in the small XML header. The binary Terse data directly follow this XML header. For positive data, compressing as an unsigned integer yields a tighter compression. For data with negative integral numbers, *TRPX* uses two-complement format for encoding, where the negative number is represented by the two’s complement of its absolute value.

Files that contain *TRPX* data can be read directly into a ‘Terse’ object, which can be decompressed by its Terse::prolix () member function. (A member function of a class is a function that has its prototype within the class definition.) The Terse::prolix () member function allows the user to specify the location where the unpacked data will be stored, by providing a container of the appropriate size, or an iterator, or memory location as the argument. If a ‘Terse’ object contains multiple images, any of these can be extracted by specifying its frame number. A ‘Terse’ object can be unpacked into any type of arithmetic data, including floats and doubles. However, when pixel values of the original data require more bits than available, they are truncated to the highest (or lowest) pixel value in the unpacked data.

#### 2.1.1. Block compression

**Figure 1:**
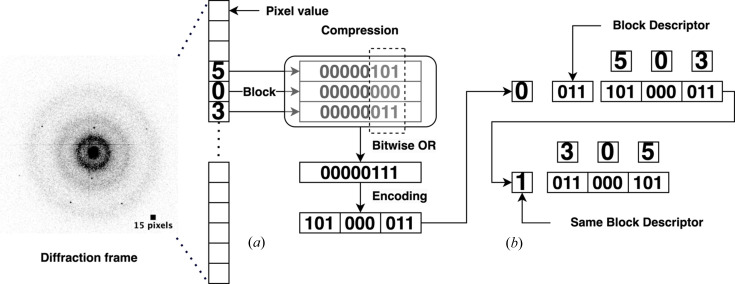
Compression scheme. (*a*) Compression of a single 16-bit 512 × 512-pixel diffraction frame. The pixel values of each data block are stripped of their most significant bits. For a block size of 3 with values 5, 0, 3 shown here, the encoded bits would be: 101 (denoting 5), 000 (denoting 0) and 011 (denoting 3). This block of data can therefore be encoded as three values of 3 bits each. The encoded 101000011 block would be pushed into the ‘Terse’ object. (*b*) Each compressed data block is described by a variable-sized block descriptor, which is preceded by a single bit. If the bit is set, the block descriptor is identical to the previous one. If the bit is not set, a new block descriptor follows. In this scenario, bits 2 to 4 define how many bits are used per value of the encoded block. If all three subsequent bits are set, the block descriptor is expanded to allow encoding of pixel values that require up to 64 bits. The black square embedded in the image represents a 15 × 15-pixel scale for reference.

*TRPX* compresses the data in fixed-size blocks (Fig. 1 ▸). The pixel values of each data block (by default 12 integral values) are stripped of their most significant bits, provided they are all either zero (for unsigned values), or all identical (for signed values). In the latter case, the sign bit is maintained.

Each compressed data block is preceded by a variable-sized block descriptor indicating the number of bits used for encoding a single pixel value. This bit depth can vary from 0 to 64. However, lower values (requiring 0 to 6 bits, corresponding to a dynamic range of 0 to 128) are more common than higher values (requiring 10 to 64 bits, corresponding to a dynamic range from 2048 to 1.8 × 10^19^).

To optimize compression, the block descriptor has a length of either 1, 4, 6 or 12 bits. The structure of the block descriptor is as follows:

*Bit 1*. If set, the previous block descriptor is used; if not, the descriptor is expanded with 3 more bits.

*Bits 2 to 4*. These indicate how many bits are used per pixel value in the encoded block. If all three bits are set, then 7 or more bits per pixel value are required, and the descriptor is expanded with an additional 2 bits.

*Bits 5 and 6*. The first 4 header bits must be 0111. The number encoded by bits 5 and 6 is added to decimal 7 to determine the number of bits used to encode each value in the block. Specifically, if bits 5 and 6 are 00, then 7 bits are used; if they are 01, then 8 bits are used; if they are 10, then 9 bits are used; and if they are 11, then at least 10 bits are used. If both bits 5 and 6 are set, the header is expanded by an additional 6 bits.

*Bits 7 to 12*. The first 6 header bits must be 011111. The number encoded by bits 7 to 12 is added to decimal 10 to determine the total number of bits used to encode each value in the block. Specifically, if bits 7 to 12 are 000000, then 10 bits are used; if they are 110110, then 64 bits are used (*i.e.* 10 + 54).

While other encoding schemes are possible, this particular one was found to be optimal for weak diffraction data and virtually indistinguishable from others for strong diffraction data. By using a variable-sized block descriptor and allowing for identical descriptors to be used for adjacent blocks, the encoding scheme can efficiently compress the data while retaining essential information.

## 3. Comparative analysis of *TRPX* compression algorithm with *gzip*, *bzip2*, CBF, *Zstandard*(*zstd*), *LZ4* and HDF5 with *gzip*, *LZF* and *bitshuffle*+*LZ4* filters

### 3.1. Test data set

Continuous-rotation electron diffraction data of an inorganic crystal were collected at PSI (Villigen, Switzerland). The diffraction experiment was carried out using a Jeol F200 transmission electron microscope with a Schottky field emission gun (FEG) and a CEOS CEFID energy filter, operated at 200 keV. The detector used was an ASI Cheetah M3 retractable hybrid pixel detector, which collected zero-loss data as 16-bit 512 × 512-pixel .tiff stacks. The data were acquired in continuous, low-gain mode at a rate of 10 frames s^−1^ while the sample was continuously rotated at 1.4° s^−1^. The unstacking of the data was performed using the *EMAN2* package (Tang *et al.*, 2007 ▸), resulting in 450 frames of 16-bit 512 × 512-pixel data. The data take up 237.8 MB of disk space.

### 3.2. Results

We evaluated the performance of several compression algorithms, including *TRPX*, *gzip* (compression levels = 6 and 9), crystallographic binary file (CBF), *bzip2*, *Zstandard*(*zstd*), *LZ4* and hierarchical data format, version 5 (HDF5) with *gzip*, *LZF* and (*bitshuffle*+*LZ4*) filters. For the CBF file format and the HDF5 library, we used the *Python Imaging Library* (*Pillow*) to rewrite the decompressed images into original .tiff format. Our current implementation of *TRPX* relies on a custom developed TIFF library to read and write .tiff files (Appendix *B* [App appb]). The evaluation was based on several metrics, including compression rate, compression and decompression speeds, and CPU utilization. Our results, obtained using a MacBook Pro with an M1 Max processor, using a single core in the case of *TRPX*, and averaged over five cycles, are summarized below.

*TRPX*. The *TRPX* algorithm reduced the data size to 38.1 MB after compression, which corresponds to 84.0% compression efficiency. The compression speed was 0.22 s user time for 450 frames, with a moderate 46% CPU utilization. The decompression speed was also fast at 0.17 s user time with a 50% CPU utilization.

*gzip*. *gzip* compressed the data to 48.4 MB, which corresponds to 79.6% compression efficiency. However, the compression speed was relatively slow at 15.6 s user time, with a high CPU utilization of 87%. The decompression speed was 1.29 s user time with a CPU utilization of 68%. When using *gzip* with the maximum compression level of 9, the resulting compressed data set size was reduced to 46.2 MB. However, this increased compression level came at the cost of a significantly longer processing time.

*bzip2*. The *bzip2* compression yielded a data size of 36 MB, corresponding to a compression efficiency of 84.8%. The compression time was slightly better than that of *gzip*, taking 12.75 s and requiring 68% CPU load. The decompression process took 4.31 s and utilized 58% of the CPU load.

*CBF*. The CBF algorithm reduced the data size to 119.8 MB after compression, which corresponds to 49.6% compression efficiency. The compression speed was relatively slow at 2.68 s user time, with a high CPU utilization of 92%. The decompression speed was 4.72 s user time with a lower CPU utilization of 52%.

*HDF5 with LZF compression filter*. The HDF5 format with *LZF* compression filter reduced the data size to 95.2 MB, which corresponds to 59.9% compression efficiency. The compression speed was 2.64 s user time with a moderate CPU utilization of 40%, while the decompression speed was 1.32 s user time, with a CPU utilization of 67%. When paired with the *gzip* compression filter, the HDF5 library compressed the data to 50.9 MB, which corresponds to 78.6% compression efficiency. The compression speed was 4.1 s user time with a moderate CPU utilization of 54%, while the decompression speed was 1.46 s user time, with a CPU utilization of 66%.

*zstd*. The *zstd* algorithm with default compression level (3) reduced the file size to 52.1 MB, which corresponds to 78.1% compression efficiency. With a maximum compression level of 19, we could reach a file size of 41.2 MB at the expense of more than 1 min (>70.0 s) user time. The decompression is faster and requires 0.76 s user time with a moderate CPU utilization of 50%.

*LZ4*. The *LZ4* compression scheme resulted in a compression efficiency of 58.8% (97.9 MB). The user time was 0.73 s for 450 frames, requiring 31% CPU load. The decompression was even faster (0.32 s) with a CPU load of 40%.

*HDF5 with bitshuffle+LZ4 compression filter*. The *bitshuffle* (Masui *et al.*, 2015 ▸) algorithm combined with *LZ4* compression implemented in *HDF5plugin* (Vincent *et al.*, 2023 ▸) resulted in a 46 MB compressed data set (80.6% compression efficiency), requiring 1.26 s user time. In this scenario, the HDF5 library scaled across multiple cores, increasing the CPU load to 175%, averaged from five trials. The decompression requires 1.24 s user time and CPU load of 163%.

#### 3.2.1. In-memory compression and decompression performance

For the evaluation of in-memory compression and decompression performance, 450 frames were loaded into memory to eliminate I/O overhead from the calculations. The compression and decompression times were then measured and averaged over 20 cycles for each algorithm (Table 1 ▸). *TRPX* had the fastest compression and decompression speeds, followed by *LZ4* and HDF5 (*LZF*). *zstd*, CBF and HDF5 (*bitshuffle*+*LZ4*) had comparable compression speeds, although the decompression speed was faster in the case of the *zstd* scheme. *gzip*, HDF5 (*gzip*) and *bzip2* had moderate performance in both compression and decompression times. HDF5 (*gzip*) was fastest among them for the in-memory compression.

**Figure 2:**
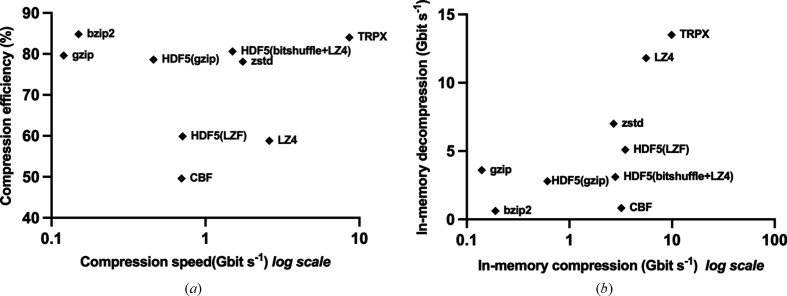
Scatter plots of benchmarked algorithms. (*a*) This scatter plot displays the compression speed (Gbit s^−1^) versus the compression efficiency (%), with each point averaged over five cycles for each algorithm. (*b*) The second scatter plot represents the in-memory compression (excluding I/O) and decompression (excluding I/O) speed, averaged over 20 cycles for each algorithm. The data on the *X* axis are presented in a log-scale format.

Based on the benchmark results, it is clear that the *TRPX* compression algorithm outperforms other compression algorithms such as *gzip*, *bzip2*, CBF, *zstd*, *LZ4* and HDF5 with *LZF*, *gzip* and *bitshuffle*+*LZ4* compression filters (Fig. 2 ▸). *TRPX*, together with *bzip2*, achieves a significantly higher data reduction rate, while *TRPX* maintains faster compression and decompression speeds. These results demonstrate the efficiency and effectiveness of the *TRPX* algorithm for compressing diffraction data, making it a valuable tool for handling large data sets in crystallography and other fields.

#### 3.2.2. Compression and decompression of cryo-EM data

The *TRPX* algorithm was not specifically designed for cryo-EM data, but is also very useful for this purpose. Over a test set of 120 short-exposure frames (the raw data prior to drift corrections), that was a dose fractionated stack using a GATAN K3 direct electron detector in counting mode (5760 × 4092-pixel arrays, 54 e Å^−2^, 40 frames s^−1^, 4 s exposure time) and occupying 5.66 GB disk space, *TRPX* achieved a compression rate of 85.4% (828.3 MB), requiring 8.6 s user time, corresponding to a compression speed of 5.3 Gbit s^−1^. Decompressing this data set was faster, and only took 2.8 s of user time, corresponding to a decompression speed in excess of 16 Gbit s^−1^. Clearly, *TRPX* scales well over many data sizes.

## 4. Discussion

The increasing availability of high-throughput data collection techniques in structural biology has created challenges in handling the massive amounts of data (Mokso *et al.*, 2017 ▸). Different compression methods have been developed to address these challenges, such as compressing signals with singularities and transient phenomena, exploiting ptychographic oversampling, or reducing data based on simple azimuthal regrouping (Ferrer *et al.*, 1998 ▸; Loetgering *et al.*, 2017 ▸; Kieffer *et al.*, 2018 ▸). The advent of *LZ4* (https://github.com/lz4/lz4), *zstd* (https://github.com/facebook/zstd), with and without combination with the *bitshuffle* algorithm, has made these tools valuable for compressing grayscale integral data (Kieffer *et al.*, 2018 ▸).

In this paper, we introduced the *TRPX* algorithm, a compression method specifically designed for diffraction data. It outperforms conventional compression techniques in terms of compression efficiency and speed. Our initial tests show that *TRPX* can almost keep up with a data stream of up to about 2000 frames (512 × 512 pixels) s^−1^ using a single CPU core on a modern laptop with Apple M1 Max System on Chip. As expected, we could verify the algorithm to be independent of the CPU type: when tested on an AMD Ryzen 9 (7900X, 12 cores, base clock of 4.70 GHz) workstation, with WD SN850X NVMe SSD and having a Linux kernel, *TRPX* compressed 450 frames in 0.18 s, while the decompression speed was 0.099 s. With extra hardware and parallel processing, *TRPX* can keep up with the fastest detectors available.

The generated *TRPX* (.trpx) files are byte-order independent, which ensures compatibility across different hardware architectures. A single .trpx file may hold many frames, in order to facilitate data management and bookkeeping. Furthermore, the compiled *TRPX* algorithm has a very small footprint and can be readily implemented in hardware, such as FPGAs (field programmable gate arrays) or ASICs (application-specific integrated circuits), allowing for potential integration with existing data acquisition systems. This integration can greatly enhance the real-time processing capabilities and overall efficiency of data collection and storage in structural biology experiments.

By providing a tailored solution to handle the specific requirements of diffraction data, the *TRPX* algorithm not only mitigates storage and transmission concerns but also facilitates more efficient data analysis and interpretation. It aims to improve the efficiency of data storage and transmission, while retaining the essential information within the diffraction data.

In conclusion, further development and widespread adoption of the *TRPX* algorithm, and its integration with user-friendly data processing and analysis tools have the potential to streamline the workflow for researchers working with high-throughput diffraction data. This can ultimately accelerate research.

## Figures and Tables

**Table 1:** Compression efficiency is calculated as the percentage reduction in file size compared with the initial size CPU utilization refers to the percentage of CPU resources utilized during compression or decompression. Compression and decompression speeds in Gbit s^−1^ relative to the original, uncompressed image size refer to ‘user time’, and exclude ‘system time’ for program initialization, memory management and I/O. (De)compression speed without I/O overhead is the ‘wall clock time’ required for in-memory (de)compression. All tests were performed on the same hardware and software setup.

Algorithm	Compression efficiency (%)	Compression speed (Gbit s^−1^)	Compression CPU utilization (%)	Decompression speed (Gbit s^−1^)	Decompression CPU utilization (%)	Compression speed without I/O overhead (Gbit s^−1^)	Decompression speed without I/O overhead (Gbit s^−1^)
*TERSE/PROLIX*	84	8.6	46	11.1	50	9.9	13.5
*gzip*	79.6	0.12	87	1.46	68	0.14	3.6
*bzip2*	84.8	0.15	68	0.44	58	0.19	0.62
CBF	49.6	0.70	92	0.40	52	3.2	0.83
*zstd*	78.1	1.75	60	2.48	50	2.7	7.0
*LZ4*	58.8	2.6	31	5.90	40	5.6	11.8
HDF5 (*LZF*)	59.9	0.71	40	1.43	67	3.5	5.1
HDF5 (*gzip*)	78.6	0.46	54	1.29	66	0.61	2.8
HDF5 (*bitshuffle*+*LZ4*)	80.6	1.5	175	1.52	163	2.8	3.1

## Appendix A Data and code availability

The *TRPX* compression algorithm, the associated TIFF library and the *ImageJ*/*Fiji* plugin (Appendix *B* [App appb]) are available in our GitHub repository (https://github.com/Senikm/trpx.git) under the MIT License. This permissive open-source license allows for the free use, modification and distribution of the software, with minimal restrictions, enabling the scientific community and other interested parties to build upon and integrate these resources into their own projects or workflows.

## Appendix B Supplementary materials

### b1. *TRPX* C++20 code

With the open-source C++ header library, integral data stored in any memory location can be compressed in memory and subsequently written to disk. Also, compressed data can be decompressed and read into any type of C++ container or memory location, storing any type of numerical values. All code is implemented in templated headers, with one header file containing the Terse-object code and two header files for XML parsing and bit manipulations.

### b2. TIFF library

As an example of usage, the distributed package also contains C++20 code for two standalone command line executables that compress, respectively decompress, TIFF files and TIFF stacks that have one grayscale intensity value per pixel, as produced by Medipix Quad and other detectors.

### b3. *Fiji* */ImageJ* plugin for *TRPX* (.trpx) format data

The *TRPX Reader* plugin is designed for reading, unpacking and visualizing image data from .trpx files in *ImageJ*/*Fiji*. The plugin reads the image data from a selected .trpx file and displays them as a grayscale image. When executed, the plugin first prompts the user to select the data file. Once the file is selected, the plugin reads its XML header to obtain important parameters required for unpacking the .trpx file. Setting the dimensions is not required and if not provided, the *TRPX Reader* calculates the dimensions from the number of pixels, assuming it is a square image. Once unpacked, the plugin creates an object with provided or calculated dimensions, populates the object with the unpacked data and displays the resulting image.
